# Peer Review of “Emergence of the First Strains of SARS-CoV-2 Lineage B.1.1.7 in Romania: Genomic Analysis”

**DOI:** 10.2196/32296

**Published:** 2021-08-13

**Authors:** Cristian Apetrei

**Affiliations:** 1 Department of Microbiology and Molecular Genetics University of Pittsburgh Pittsburgh, PA United States

**Keywords:** infectious disease, COVID-19, strain, virus, Romania, transmission, spread, mutation, impact, case study, genome, sequencing, genetics, epidemiology, variant, virology, lineage

*This is a peer-review report submitted for the paper “Emergence of the First Strains of SARS-CoV-2 Lineage B.1.1.7 in Romania: Genomic Analysis.”*

## Round 1 Review

### General Comments

This paper [1] reports the identification and characterization of the SARS-CoV-2 B.1.1.7 variant (the English variant) in North-East Romania and a synopsis of the circulation of this variant in Romania. The manuscript is timely, straightforward, and professionally crafted, and the results are of interest for the characterization of SARS-CoV-2 strains. Such routine surveys are necessary to trace the emergence of new variants of interest and are scarce in Eastern Europe.

### Minor Comments

The manuscript needs some revision of English. It is generally well prepared, but there are several instances in which it could benefit from a professional revision.
